# List of Accidents, Admitted into the Pennsylvania Hospital, from August 8th to August 22d, 1838

**Published:** 1838-08-29

**Authors:** J. Forsyth Meigs

**Affiliations:** Resident Surgeon


*List of Accidents, admitted into the Pennsylvania Hospital, from August 8th to August 22d,* 1838.

[Reported by J. FORSYTH MEIGS, M. D., Resident Surgeon.]

A case of sprain of both wrists, from lifting a heavy weight. When admitted, there was considerable swelling and inflammation about both wrists; arms placed upon splints, elevated, and lead water kept constantly applied; since discharged cured.—A case of lacerated wound of fingers, caused by the explosion of a charge while blasting rocks. There was compound comminuted fracture of the left malar bone, and of the left side of the lower jaw, by which latter the fluids taken into the mouth were hastily discharged; the globe of the left eye was completely collapsed, there being also a fracture of the left orbicular process of the frontal bone. The last joints of the fore and middle fingers of the left hand were opened and the integuments much torn. When admitted, the man was sensible, and could answer questions, though very indistinctly; did not complain of much pain, and had not lost much blood; some small pieces of the malar bone and of the lower jaw were removed; the wounds slightly drawn together by adhesive straps, and dressed with cold mucilage; the hand poulticed and placed upon a splint; the patient took an opiate; for diet liquids. Died in four days of arachnitis and effusion into the cranium.—A case of compound fracture of both bones of the left leg, caused by a rail-road car passing over it. External wound six inches in length, with the internal belly of the gastro-enemius muscle protruding; when admitted the man was inebriated, and of very bad habits of body; hæmorrhage not great; one small vessel secured; wound brought together by strips; fracture reduced, and leg placed in fracture box. In a short time he was attacked with mania a potu, for which he took opium and brandy; had a blister to the back of his neck; he became very much prostrated; the whole leg mortified, and secondary hæmorrhage coming on, bran was placed about it, which arrested the hæmorrhage. Died four days after admittance.—A contusion of left hand, caused by the falling of a heavy stone upon it; when admitted the hand was very much inflamed and very painful; placed on a splint, and cloths kept constantly wet with lead water applied; a small collection of matter formed, which was discharged by an opening; since discharged cured.—A case of incised wound of the back of the neck from a blow with a saw; dressed with adhesive strips, and kept quiet. From its partaking of the nature of a lacerated wound, did not unite by the first intention, but is now filling up by granulation.—A case of compound dislocation of the left ankle, with a simple fracture of both bones of the right leg, and a badly lacerated wound of the left thigh, by which the integuments were stripped from half the surface of the thigh, caused by a rail-road car passing over the boy. Death in a few hours.—A case of lacerated wound of the right hand and arm, from being drawn in between two rollers; a compound comminuted fracture of the middle ring fingers; first and second metacarpal bones entirely separated; the thumb very much injured; its tendons being laid bare, and an extensive flap of the integuments of the forearm torn up. Wound dressed with the adhesive strips; dry lint applied, and then placed on a splint. The next morning, after a consultation, Dr. NORRIS amputated the two fingers at the middle joint; parts at present sloughing to considerable extent; dressed with poultices; full diet.—A case of severe contusion of the loins in a boy of six years, caused by a horse treading upon him. When admitted, a tumour, the size of a large orange, had formed in consequence of the effusion of blood; boy placed in bed; kept quiet, and lead water applied; some inflammation took place, which was relieved by cupping, and a smart purge; at present reducing in size.—A case of compound fracture of the last phalanges of the first and second toes of the right foot, from a heavy bar of rail-road iron falling upon it. External wound extends half-way round the great toe; but little hæmorrhage; dressed with adhesive strips, a roller, and the fracture box; kept constantly wet with lead water by means of a syphon; dressings removed in four days, when the wound in the great toe was nearly united; second toe sloughing, and the last phalanx of which has since been removed; at present dressed with strips and cerate; doing well.—A case of fracture of the acromium, from a severe contusion; treated by clavicle apparatus; since discharged.—A case of contusion of knee and ankle from a dray passing over them; leg placed in fracture box, and lead water applied; doing well.—A case of compound fracture of the skull from a blow with the handle of a windlass; patient totally insensible; there was an external wound three inches long; a comminuted fracture of the superior middle portion of the frontal bone, the pieces being driven in upon the brain, two or three drams of which escaped from the wound, and an extensive depression on the left inferior portion of the same bone. Death in a few hours.—A case of simple transverse fracture of both bones of the leg, from the rolling of a hogshead against it; dressed with fracture box as usual; doing well.—A case of hæmatacele, caused by a severe strain; admitted about six hours after the accident, when there was a large tumour in the left side of the scrotum, evidently from an effusion of blood into the cavity of the tunica vaginalis; leeched and supported in bag truss; lead water applied; doing well.

